# Supplementary material for: Patterns of Intron Gain and Loss in Fungi
Source: PLoS Biol. 2004 Nov 30;2(12):e422. doi: 10.1371/journal.pbio.0020422 (PMC532390; doi:10.1371/journal.pbio.0020422)
Supplement: Table S1 — Also available at http://genes.mit.edu/NielsenEtAl/. (4.3 MB ZIP). [file pbio.0020422.st001.zip › NielsenEtAl/html/13.html]

AN7750.1.NCU06389.1.MG06138.1.FG06212.1


```
 CLUSTAL W (1.82) Multiple Sequence Alignments - Introns Inserted


Sequence 1: MG06138.1	845 aa
Sequence 2: FG06212.1	844 aa
Sequence 3: NCU06389.1	911 aa
Sequence 4: AN7750.1	878 aa
Alignment Length: 1009 aa
Number Identitical Residues: 237 aa
Alignment Score (without introns) 13510


MG06138.1 	MTQPLPPQPTDKRRVKVYELRNNEWYDRGTGFCTAAYIP0IS~DSPDEDTPREP~RVIVE
NCU06389.1	--------------------------------MTGSIAV~PA~SAP------PN1TLIVW
FG06212.1 	MAQPVPHQPTDRKRVKVYELRNNDWFDRGTGFCSASFET0LD~DGR-----KDP~RVVVE
AN7750.1  	MALELQP-PSDRKRVKVYELKENDWFDRGTGFCTG--QI~LD0D--------EP~RIFVE
          	 :      .:.    .  .  ...  . .:.  :.        .            :.* 

MG06138.1 	SEDHPDRVLLDVRICKEDGFQQQQ-~---------------------GM2K---~-~---
NCU06389.1	TENNVDMALS---------FQEAE-~---------------------GC~AMIW2K~---
FG06212.1 	SEDHPDRLLLETKIQKEDGFQKQQ-~---------------------GT~STCL~I2YPD
AN7750.1  	SEDKPNRVLLETKISKDGGYQKQQD1TLIVWTEPNQTDMALSFQEAEGC~AMIW2N~---
          	:*:: :  * .    ....:*: :. :    :....:. : : ..:.*            

MG06138.1 	FVSGVQS------------SFRGQM~A-HD1DSLSDDLALDTGP---SLPPPELASLPEL
NCU06389.1	FIDGVQQ------------TFHNVL~GGAD~DSLSEDLTMDMSNSTFTLPPADLANLPDL
FG06212.1 	CVAGTQQWCRYGIELPGSRRMRNNL0A-FY1DGLSDDLAIDVPPA-INLPTAELGNLGDI
AN7750.1  	FVNSVQQ-------------HLLTL~AAGD1DALSEDLESMHSVT---LPAPELANLPDI
          	 : ..*.                 : ..   *.**:**      :   **..:*..* ::

MG06138.1 	EHSMRMMASSNTGREALTKCIMSEDYIGRLIPLVEMAEDLESLDDLHRLCNIMKTILLLN
NCU06389.1	ENIMRQMSNTANGREMLVKSIMEHDIIPSLGNLVEMAEDLESLDDLFRLCNMTKTMLLLN
FG06212.1 	ESTMRMMSTTANGRDALAKAIMAEDFIGKIVPLVEMAEDLESLQDLHRLCNIMKTVILLN
AN7750.1  	DHLMRAASMAQTGRDALSKCVIRDDYIQKLLPLVTVAEDLESLPDLHRLCNIMKSLILLN
          	:  **  : : .**: * *.:: .* *  :  ** :******* **.****: *:::***

MG06138.1 	DSGIIEHAVSDECIEGVVGALEY~DPDFPSHKANHRQWLKNRSRYIEVVKLQDPAISRKV
NCU06389.1	DTAIIEKAVSDECVLGVVGALEY~DPDFPKHKANHRQWLSNQTRFKEVVKIEDDTIRRKI
FG06212.1 	DTSIIEHAVSDECVLGVVGALEY~DPDFPSHKANHRHWLGNQGRYKEVVPIEDEQIRRKI
AN7750.1  	DTTIIETVVTDSVILGVVGALEY1DPEFPTHKANHRQYLSDESRYKEVVPIRDETIRRKI
          	*: *** .*:*. : ******** **:**.******::* :. *: *** :.*  * **:

MG06138.1 	HQTHRLQYLKDVVLARILDDPTFSVLNSLIFFNQVDIVNHLGQDPELLMELFGKFTDPSV
NCU06389.1	QQTFRLLYLKDVVLARILDDPTFSVLNSLIFFNQVDIINHIQTNGGFLNDLFSIFADPSA
FG06212.1 	HQTYRLQYLKDVVLARILDDPTFSVLNSLIFFNQVDIVQHLQANAAFLNELFGIFSLSNN
AN7750.1  	RYTWRLQYLKDVVLARILDDPTFSVLNSMIFYNQVDIVNHIQSDGAFLKELFSVFDPRSA
          	: * ** *********************:**:*****::*:  :  :* :**. *   . 

MG06138.1 	EPLRQKEALQYIQQCCAIAKNLQPPIRQLLYNSFITHGLLHVINFGLRQPDVTARVGATD
NCU06389.1	QHLRKKQAVTFLHDCCAIAKNLQPPARQNLYTNFLGHGLVRVINFGLCDHDVTVRVHATD
FG06212.1 	DQKRKKEAVLFIQQCCAIAKNIQPPARQTLYNNFLAHGLLQVINFGLRHHDVGVRVGATD
AN7750.1  	DAKRKEDAVQFLHQCASIAKNLQVPERATLFANLISHGLFAVIAFAIKHPNPAIRTTGID
          	:  *:::*: ::::*.:****:* * *  *: .:: ***. ** *.: . :   *. . *

MG06138.1 	ILVSMIDHDPQMVRQTIYRQIHENQPPLTDALIDLLLAEVDLGIKSQISDALKVLLDPNP
NCU06389.1	ILVAMIDHDPQMVRHTIYRQLQERQKPLTDVIIDLLLVEVDLGVKSQMQDALRILLDVAP
FG06212.1 	IMISIIDHDPQMIRQTLYRQVHEKVTPLTDSLIDLLLVEVDLGVKSQISEALKVLLDPGP
AN7750.1  	ILVALLDHDPIMMRSYMLKAVNEKKTPLTDTLIDLLHTETDLGVKNQLADAIKVLLDPQV
          	*:::::**** *:*  : : ::*.  **** :**** .*.***:*.*: :*:::***   

MG06138.1 	PPMP-PDASKPNGEPLPRR--PQGPDPEQDGFLQHFYDHSAARLFRPLLELEGKTVL~K-
NCU06389.1	IMPPDAREYPPNQQRLRQL---QSIDPQQELLIQHFYENSAARLFKPLLDLEGQTEL~K-
FG06212.1 	QQLQNENFQKANGELYNEEMLQTSTDPRQEVFLHAFYEQSAPKLFKPLVDLEKRTDM~N-
AN7750.1  	PIQAAMARAGPEAAKVRPN-------ILSDAFVQNHFEESSRRLFAPLKRLEDQSSP1HG
          	          .:                .: ::: .::.*: :** **  ** ::   :.

MG06138.1 	FSVADDGVFNYLNDILCFYIRQHMHRCKLFILQNNISQRFLQLLACDQKHVQLE1SLV~A
NCU06389.1	FQPMQEYIFGYLNDILCSFIRQHQHRAKYFLITHSLAQRFVQLLSCKQKHLQ--~-LV1A
FG06212.1 	FSVQQASMFTYLIEILTFFIRQHTLRVKFYVMNNDIAKRVSQLLGCPEKFLR--~-LV1A
AN7750.1  	LTFQAVALYSHLVDILTFFVRQHLYRCRNVIQNERLAAHITQLLRVPQKHLK--~-LT1A
          	:      :: :* :**  ::***  * :  :  . :: :. ***   :*.::    *. *

MG06138.1 	IRFFRQLIALKDEFWIRQIAEKQVLGPILDVLIGVLPRDNLLGSACLDVFAFIHQESIKD
NCU06389.1	IRFIKQLVLMNDEFYMKHIAEKQILGPILDVLLRAITRDNLLCSACLDLFVLINKENVKD
FG06212.1 	IRFFRSIIGMQDEFYIKHLTEKQVLGPILEVLIGTMPRDNLLASACLEFFEFIKKENVKD
AN7750.1  	LKFFRTLLSLQDTFYQALMTHNDTFGLILDIVYETMPRDNLLNSACLELFEFIKREHIKP
          	::*:: :: ::* *:   ::.:: :* **:::  .:.***** ****:.* :*::* :* 

MG06138.1 	LARHLVETHREKVQFLTCLSLFRDILDMYDKPQGNYSATIDGYFYDSEES-----VLVPR
NCU06389.1	LIKHLVENYREKIMALSHMDTFREMVSRYDQTEG-YTTNPEYFMDEDEDEGPTAAESERR
FG06212.1 	VIKYLVVNHRDQLMTLSYMPTFRDIVLRYDQTRG-YTSNVD-YFMEGED------EMGRR
AN7750.1  	ITLHVVGKYGDKLRNITYVDTFQGLILHYEQLQG-YSEEGDSTLYSQEES-------TPG
          	:  ::* .: :::  :: :  *: ::  *:: .* *:   :  : . *:.          

MG06138.1 	PNPANTRGMMEHLAVDPDEEAFWNGSD-EEE~ELQTRAGP----PLQVSGNGAVGPKPLV
NCU06389.1	PTRPTSGLMEEQLTVDQAQEEYWNGVSDEED~EGPQNDSH----RTNGTATTLPLLKPLV
FG06212.1 	P-PPNTR-LMEQITVDQEQEEYWAGSDNEEE~EDNSSKEG----EKTPSTNGSISSKLLV
AN7750.1  	LKMQPTGRWQGVKEMDPAEEEYFNTSDDEEE0WQQDTQASGTLLSQVPNGAASPSVKSLV
          	 .   :        :*  :* ::   ..**:         .:      .       * **

MG06138.1 	DYASDDDVDE-NGDTEVKPTPSVEVDE-----AKAVVSEDDSEGPVWTPTSSVGPPPERL
NCU06389.1	EYGSDDEDGV-AGDEATAPAKKDDEDNSAKSNGEPPVTPTKGGPATAAAAAVVATPPERI
FG06212.1 	DYASDEETDE-NADPEATPG------------SHSDVASDAVGSPIDSSLGGGVPPPERL
AN7750.1  	DYPDDDEEDVNAMDTKPEPDTAQKQLQPAEASNEDAPADPSIEVPSTPPSQVVQTPPERL
          	:* .*:: . .  *    *    .  ..:.:. .   :      .  ..     .****:

MG06138.1 	SEKRRREEDEED-EIDKLMQTNKRRNSSSATMKP--------------------------
NCU06389.1	SEKRRREEDEDDDAFDKLLQ-SKRRGSNASAASAGADIRKKMGISEDDGPAAEGATTEDN
FG06212.1 	SEKRRREEEEDDDEIGKLMH-NKRRNSS-GSISS--------------------------
AN7750.1  	SEKRRREE-EDDDELVKLTSGPKRRSSTSSSTGSAGFLRKK-------------------
          	******** *:*. : **    ***.*.:.:  ...   ..                   

MG06138.1 	----ATGLAAGPVRRSRSLTGRDSPSNGNKKIAISISPALKLATTG-----------QPV
NCU06389.1	GTSMADRTSPNPASPLAAATAVTTSLSTPKKIAFNISSKVKSMLGGGGGGKPSEPEADPN
FG06212.1 	----VSSNPRGLTKR-KSFTASSS-SGSSKKIAISLSSGLKTGSG-------------PG
AN7750.1  	-RSGSVGSVSVPNKVAQGDGRPSTGNAVPKRIAINLSSSVKSRRES------------RN
          	  :              .     :     *:**:.:*. :*    .              

MG06138.1 	SEDGSE------------------------------------------------------
NCU06389.1	TTTTSANDKAQETSITTGATTASGKENVRIGEREEKEEGKETSKSVVQEVEGEEEEDPNA
FG06212.1 	SNEES-------------------------------------------------------
AN7750.1  	ATGCCIKCGWERT-----------------------------------------------
          	:   . .   . :                                               

MG06138.1 	---
NCU06389.1	MKD
FG06212.1 	---
AN7750.1  	---
          	
```
